# Cinnamaldehyde-Contained Polymers and Their Biomedical Applications

**DOI:** 10.3390/polym15061517

**Published:** 2023-03-18

**Authors:** Guangyan Zhang, Tianlong Li, Jia Liu, Xinran Wu, Hui Yi

**Affiliations:** 1 School of Materials and Chemical Engineering, Hubei University of Technology, Wuhan 430068, China; 2 Tongji Medical College, Huazhong University of Science and Technology, Wuhan 430022, China

**Keywords:** cinnamaldehyde, reactive oxygen species, stimuli-responsive, drug delivery

## Abstract

Cinnamaldehyde, a natural product that can be extracted from a variety of plants of the genus *Cinnamomum*, exhibits excellent biological activities including antibacterial, antifungal, anti-inflammatory, and anticancer properties. To overcome the disadvantages (e.g., poor water solubility and sensitivity to light) or enhance the advantages (e.g., high reactivity and promoting cellular reactive oxygen species production) of cinnamaldehyde, cinnamaldehyde can be loaded into or conjugated with polymers for sustained or controlled release, thereby prolonging the effective action time of its biological activities. Moreover, when cinnamaldehyde is conjugated with a polymer, it can also introduce environmental responsiveness to the polymer through the form of stimuli-sensitive linkages between its aldehyde group and various functional groups of polymers. The environmental responsiveness provides the great potential of cinnamaldehyde-conjugated polymers for applications in the biomedical field. In this review, the strategies for preparing cinnamaldehyde-contained polymers are summarized and their biomedical applications are also reviewed.

## 1. Introduction

Natural products are renewable resources and widely present in nature. They are produced by a variety of natural sources, such as marine organisms and plants, and include both complex mixtures (e.g., plant essential oils) and small-molecule compounds (e.g., amino acids). According to the production route of natural products, they can be divided into primary or secondary metabolites [1]. For example, plant essential oils are mainly composed of an array of secondary metabolites produced by plants to cope with external environmental stressors (e.g., ultraviolet radiation and unfavorable pH) or to defend against the invasion of pathogenic microorganisms (e.g., bacteria, fungi, and viruses) [2].

Cinnamon essential oil, one of the most popular researched plant essential oils, can often be extracted from various plant parts (e.g., bark and leaf) of several trees of the genus *Cinnamomum*, and has been applied in food, cosmetics, and other fields due to its unique aroma and excellent antibacterial activity [3,4,5]. However, the complex composition of cinnamon essential oil makes it difficult to control the consistency of its composition and bioactivity, thus limiting its application. It is worth noting that cinnamaldehyde is an important component of cinnamon essential oil, although the composition of cinnamon essential oils vary depending on the cinnamon species and the plant parts from which it is extracted. In some cinnamon essential oils, such as those extracted from *Cinnamomum zeylanicum* and *Cinnamomum cassia*, the content of cinnamaldehyde can be as high as about 90% [6].

Cinnamaldehyde, denoted CA, has already been reported to be one of the most active compounds in cinnamon essential oils, with various biological activities. The reported biological activities of cinnamaldehyde mainly include insecticidal [7], antibacterial [8,9,10,11], antifungal [12], antioxidant [13], anti-hyperglycemic [14], anticancer [15,16], and other bioactivities [17]. Therefore, cinnamaldehyde has attracted widespread and great interest in various fields, especially in the fields of food [18] and biomedicine [13]. For instance, in 2021, Thirapanmethee et al. reported that cinnamaldehyde showed potent antibacterial activity against clinically isolated multidrug-resistant (MDR) Acinetobacter baumannii strains from various sources (e.g., sputum, urine, and blood) with low minimum inhibitory concentrations ranging from 0.01–0.04% (*v*/*v*), as well as some synergistic effects when applied with other antibiotics. The results suggest that cinnamaldehyde may be an alternative to control infectious diseases [8]. Moreover, cinnamaldehyde is also a reactive oxygen species (ROS) generation agent, and thus can be used to induce tumor cell apoptosis by promoting intracellular ROS production [19].

In 2022, the results of a phase I clinical trial of cinnamaldehyde for the treatment of fungal infections caused by *Candida* spp. revealed that the three ointments evaluated were proved to be safe and tolerable with a reduction of >99% *Candida* spp. CFU (35 individuals; orabase ointment containing 200 µg/mL, 300 µg/mL, or 400 µg/mL cinnamaldehyde; 3 times a day; 15 days) [20]. Another randomized, double-blind clinical trial for the treatment of minor recurrent aphthous stomatitis showed that cinnamaldehyde mucoadhesive patches were effective in reducing aphthous lesions and pain intensity in patients (44 individuals) [21].

**Figure 1:**
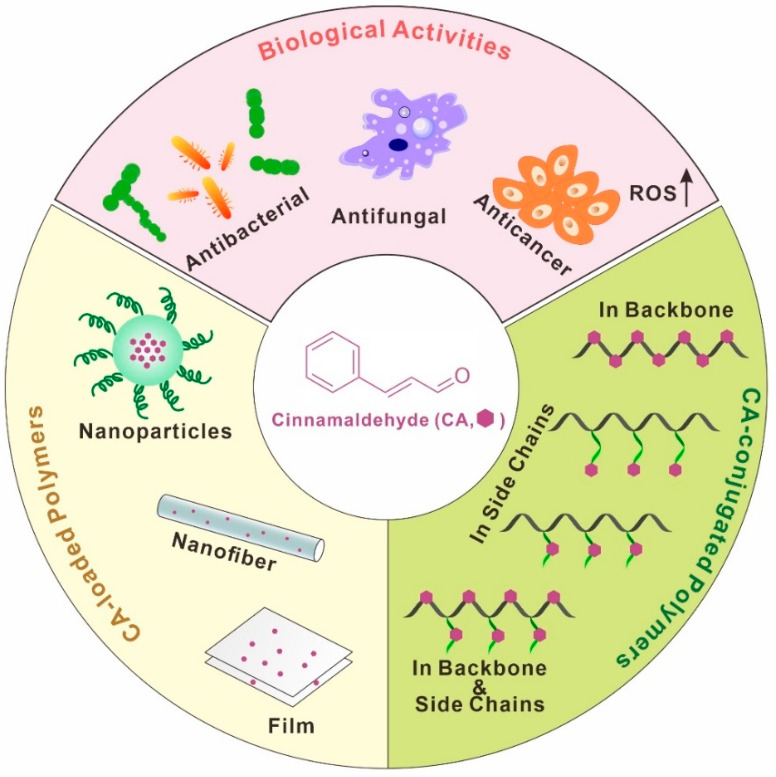
Schematic diagram of the main biological activities of cinnamaldehyde and cinnamaldehyde-contained polymer.

However, the poor water solubility (approximately 1.1 g/L at 20 °C), sensitivity to light/air, and allergic reactions of the skin of cinnamaldehyde limit its applications. In order to overcome the aforementioned disadvantages, one strategy is to develop a sustained or controlled-release polymeric system for loading or encapsulating cinnamaldehyde [22,23]. Since there is no chemical reaction between the loaded cinnamaldehyde and the polymers used for encapsulation, the resulting complex is referred to as “(1) cinnamaldehyde-loaded polymer” in this review (Figure 1).

The other strategy to fully exploit the efficacy of cinnamaldehyde is to develop novel cinnamaldehyde derivatives [24]. For example, Tang’s group designed and synthesized a tryptamine–cinnamaldehyde twin drug with an acid-cleavable linkage, and tryptamine–cinnamaldehyde can be emulsified to form nano-prodrugs for targeted synergistic glioma therapy [25]. Chen et al. linked pimonidazole (decreasing intracellular glutathione (GSH) level) and a pH-responsive cinnamaldehyde derivative (acetal between cinnamaldehyde and tris(hydroxymethyl)ethane, increasing ROS level) to lysine to enhance the efficacy of tumor therapy [26]. Besides small-molecule cinnamaldehyde derivatives, polymers containing cinnamaldehyde moieties also play important roles. Since cinnamaldehyde is involved in chemical reactions, the resulting cinnamaldehyde-derived polymer is referred to as “(2) cinnamaldehyde-conjugated polymer” in this review.

Although some papers have reviewed the research progress of cinnamaldehyde, cinnamaldehyde-derived compounds, and cinnamaldehyde analogs as antifungal [27], antibacterial [28], and therapeutic agents [16,29] in the food and medical fields, there is no comprehensive review summarizing the recent developments of “cinnamaldehyde-contained polymers”. In this paper, we will provide an overview of the up-to-date developments in cinnamaldehyde-contained polymers. First, the preparation strategies of (1) cinnamaldehyde-loaded polymers and (2) cinnamaldehyde-conjugated polymers will be introduced. Next, the applications of cinnamaldehyde-contained polymers in the biomedical fields will be reviewed. Finally, the potentials of cinnamaldehyde-contained polymers will be discussed.

## 2. Preparation Strategies of Cinnamaldehyde-Contained Polymers

### 2.1. Cinnamaldehyde-Loaded Polymers

Due to the rich biological activities of cinnamaldehyde, cinnamaldehyde is usually directly encapsulated in polymeric films, microspheres, liposomes, or nanoparticles as a functional component to improve the performance of polymers, such as antibacterial properties. Up to now, melt extrusion or pressing, solvent casting, coacervation, electrospinning, emulsion–solvent evaporation, and other methods have been reported to load cinnamaldehyde into various polymers. The reported cinnamaldehyde-loaded polymers have been summarized in Table 1.

Poly(lactic acid) (PLA) and chitosan (CS) were the most investigated polymers for loading cinnamaldehyde. Poly(lactic acid), a kind of polyester that can be derived from renewable resources, is one of the most promising bio-based polymers due to its excellent biodegradability and has been applied as a packaging film in the food field or as an anti-adhesion film in the medical field [30,31,32,33,34]. In order to improve food safety, cinnamaldehyde can be incorporated into PLA film via solvent casting or compression molding technology to enhance the antioxidant, antifungal, and antibacterial activities of food packages, thus extending the shelf life of packaged food such as bread and fruits. Additionally, PLA can also be mixed with other substances (e.g., zein [35], poly(butylene adipate-co-terephthalate) [36], and starch [37]) to prepare CA-loaded monolayer or bilayer polymeric films with good antimicrobial activity. Poly(vinyl alcohol) (PVA) [38,39] and low-density polyethylene (LDPE) [40] are also investigated for preparing cinnamaldehyde-loaded polymeric films.

Particulate carriers (e.g., microspheres and nanoparticles) are another popular studied way to incorporate cinnamaldehyde due to their advantages in additivity. For instance, Yeldir et al. [41] prepared cinnamaldehyde-loaded chitosan microspheres successfully by dropping cinnamaldehyde/chitosan mixture solution (in 7% acetic acid, *v*/*v*) slowly into NaOH solution (10%, m/v) with an insulin syringe, avoiding the use of surfactants or crosslinkers. Subhaswaraj et al. [42] prepared cinnamaldehyde-loaded chitosan nanoparticles with a mean diameter of around 200 nm by an ionic gelation method using pentasodium tripolyphosphate (negative charge) as a crosslinker. In addition, casein and poly(DL-lactide-co-glycolide) (PLGA) have also been investigated as particulate carriers for encapsulating cinnamaldehyde.

**Table 1:** A Summary of Cinnamaldehyde-loaded Polymers.

Polymer	Type	Preparation Methods	Feed Ratio of CA	EE ^1^/LC ^2^	Investigated Biological Activities			Ref.
					Activities	Testing Objects & Methods	Results	
Poly(lactic acid) (PLA)	Film	Solvent casting	10–50% (CA/PLA, *v*/*w*) CA/β-CD inclusion: 5–30 wt% of PLA	- CA/β-CD inclusion: 63.2% ^1^/6.46% ^2^	Antibacterial Antibacterial	Disk diffusion assay: (1) *S. aureus* (2) *E. coli* (1) *L. monocytogenes* (2) *E. coli*	Inhibition zone: (1) 14–50 mm (2) 8–20 mm (1) 60.6% (0.323% CA) 100% (>=0.646% CA) (2) 37.4% (0.323% CA) 100% (>=0.646% CA)	[32] [33]
			5–10 wt	96–97% ^1^	Antioxidant	DPPH method	>90% (after 3 h in DPPH solution, 10 wt% CA)	[34]
Zein/PLA	Film	Solvent casting	1–5% (CA/film solution, *v*/*v*)	-	Antioxidant Antibacterial	DPPH method ABTS method Disk diffusion assay: (1) *E. coli* (2) *S. aureus*	11.6–32.3% 0.7–12.9% (1) 11.75 mm (3%), 15.76 mm (5%). (2) 2.29 mm (3%), 12.67 mm (5%).	[35]
PBAT/PLA	Film	Twin-screw extrusion	2–10 wt%	-	Antifungal	Disk diffusion assay: (1) *Penicillium* sp. (2) *Aspergillus niger* (3) *Rhizopus* sp.	Inhibition zone: (1) 3.44–5.85 mm (2) 2.68–4.15 mm (3) 2.96 mm	[36]
Starch/PLA	Bilayer Film	Compression molding	0.2 g/g PLA film	0.117 g/g PLA film ^2^	-	-	-	[37]
Poly(vinyl alcohol) (PVA)	Film	Solvent casting	150–600 µL/g film	1.55–12.47 µL/g film ^2^	Antibacterial	(1) *B. subtilis* (2) *E. coli*	Inhibition: (1) 67.2% (1.55 µL/g film) 100% (5.59 µL/g film) (2) 29.2% (1.55 µL/g film) 100% (5.59 µL/g film)	[38]
Starch/PVA	Film	Solution casting	0.4/4/4 CA/starch/PVA, *w*/*w*/*w*	-	Antimicrobial	Disk diffusion assay: S. *putrefaciens*	Inhibition zone: 10.78 mm	[39]
Low-density polyethylene (LDPE)	Film	Melt Pressing	0.2/0.8 CA/β-CD, *w*/*w*	76–91% ^1^ (Affected by stirring speed: 250–1000 rpm)	Antifungal	*B. cinerea*	Inhibition: 25.4% (1 wt%, pure CA) 99.9% (5 wt%, pure CA) 10.9% (1 wt% *, CA/β-CD)	[40]
Chitosan (CS)	Microspheres	Dropping CA/CS solution into NaOH solution	25% 50% CA/CS, *w*/*w*	184 mg/g CS ^2^ 350 mg/g CS ^2^	-	-	-	[41]
	Nanoparticles	Ionic gelation method	(1) 0.5% (*v*/*v*) (2) 0.4–2.4 µg/mL	(1) 65.04% ^1^ (2) 7.47–27.42% ^1^	Antibacterial Antioxidant	*P. aeruginosa PAO1*; ABTS method	MIC: 1000 µg/mL 9.24–21.76% 17–39.2% (pure CA)	[42] [43]
	Liposomes	Ethanol injection method & CS decorating	CA-loaded liposomes: 0.1/0.8 CA/lecithin, *w*/*w*	38–52% ^1^ (Affected by CS concentration: 0–4 mg/mL)	Antibacterial	*S. aureus*	MIC was affected by CS concentration: MIC (CS concentration) 200 μL/mL (0 mg/mL) 12.5 μL/mL (4 mg/mL)	[44]
Poly(DL-lactide -co-glycolide) (PLGA)	Nanoparticles	Emulsion freeze-drying method	45 μL/50 mg/20 mL CA/PLGA/H_2_O, *v*/*w*/*v*	2.36 mg/mL ^2^	Antifungal	*C. albicans*	MIC: 250 μg/mL (CA-PLGA) 32.7 μg/mL (pure CA)	[45]
PLGA-PEG	Nanoparticles	Nanoprecipitation method	6.6/0.4/10 (CA/DATS/PLGA-PEG, *w*/*w*/*w*)	1.0% CA + 1.5% DATS ^2^	Anticancer	Breast cancer cells: (1) MDAMB-231 (2) MCF-7	The best synergistic effect for killing (1) MDAMB-231: 37.5 μM CA + 40.0 μM DATS Inhibition: 50.6% (2) MCF-7: 100 μM CA + 50 μM DATS Inhibition: ~50%	[46]
Casein	Agglomerates	Coacervation method	30% (CA/casein)	86.5% ^1^	Anticancer	Lung cancer cells: A549 NSCLC	IC50: 7.65 μg/mL * 45.89 μg/mL (pure CA)	[47]
Gellan/PVA	Nanofibers	Electrospinning	3 nanofibers: 1 mg/mL (NF1000); 2.5 mg/mL (NF1200); 5 mg/mL (NF1400)	17.3 ± 4.1% ^1^	Anticancer	Breast cancer cells: MCF-7	Inhibition: 20–27.7% (Nanofibers); 44.5% (pure CA)	[48]
					Antimicrobial	(1) *C. glabrata;* (2) *C. albicans;* (3) *S. aureus;* (4) *P. aeruginosa*	Inhibition: (1) 71%, 88%, 89% (2) 40%, 50%, 49% (3) 69% (NF1400, 60 min) (4) 59% (NF1400, 60 min)	
Gelatin/PVA	Nanofibers	Electrospinning	13/0.5/2.6/0.2 PVA/GEL/CA/FLU, wt%	CA: 73.84% ^1^ FLU: 68.58% ^1^	Antifungal	*C. albicans*	Inhibition:	[49]
Polypropylene (PP)	Matrix	Melt extrusion	6 wt% (CA/O-ZnO)	28.3% ^1^; 4% CA/O-ZnO in PP ^2^	Antibacterial	(1) *S. aureus;* (2) *E. coli*	Inhibition: (1) 70.9% (2) 75.6%	[50]

Note: * Calculated based on the percentage of CA in the targeted polymers. Abbreviations: EE = Entrapment Efficiency; LC = Loading Capacity; β-CD = β-cyclodextrin; PBAT = Poly(butylene adipate-co-terephthalate); PEG = Poly(ethylene glycol); DATS = diallyl trisulfide; FLU = fluconazole; DPPH = 2,2-Diphenyl-1-picrylhydrazyl; ABTS = 2,2′-Azino-bis(3-ethylbenzothiazoline-6-sulfonic acid) diammonium salt; *S. aureus = Staphylococcus aureus; E. coli = Escherichia coli; L. monocytogenes = Listeria monocytogenes; B. cinerea = Botrytis cinerea; B. subtilis = Bacillus subtilis; C. albicans = Candida albicans; C. glabrata = Candida glabrata*. For column EE^1^/LC^2^, the data with ^1^ represent EE, and the data with ^2^ represent LC.

Besides film and particulate carriers, nanofiber prepared by the electrospinning method can also be used for incorporating cinnamaldehyde. Sometimes, cinnamaldehyde formed an inclusive complex with β-cyclodextrin (β-CD) first, and then the obtained cinnamaldehyde/β-CD inclusion was used to prepare cinnamaldehyde-loaded films [33,51] or nanofiber via electrospinning [52].

### 2.2. Cinnamaldehyde-Conjugated Polymers

Due to the presence of the highly reactive α, β-unsaturated aldehyde group, cinnamaldehyde can also react with various functional groups such as primary amine. Thus, cinnamaldehyde can be coupled to the side chains of polymers, act as a monomer for polymerizing with other monomers to form the backbone of polymers, or react as a bridge to connect two polymer segments. The reported cinnamaldehyde-conjugated polymers have been summarized in Table 2.

**Figure 2:**
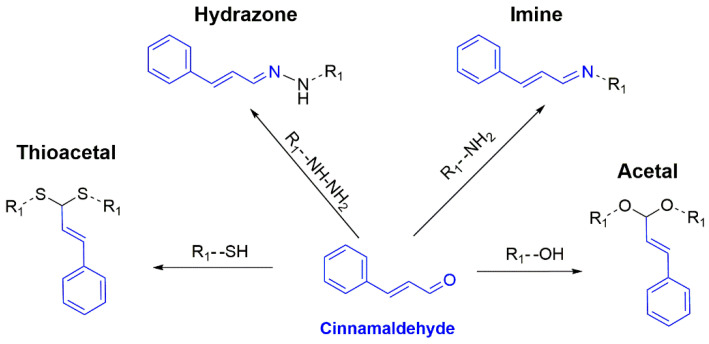
Chemical structures of typical stimuli-responsive linkages between cinnamaldehyde and polymers. Note that R_1_ represents a polymeric chain.

To realize the controlled release of cinnamaldehyde, cinnamaldehyde-conjugated polymers are commonly synthesized using stimuli-responsive linkages such as imine, hydrazone, acetal (pH-responsive), and thioacetal (ROS-responsive) as shown in Figure 2. The introduction of stimuli-responsive linkages not only enables the controlled release of cinnamaldehyde, but also gives stimuli responsiveness to the polymer. For example, under an acidic environment, the acid-cleavable bonds break, which changes the hydrophilicity and hydrophobicity of the polymer while releasing cinnamaldehyde, ultimately affecting the self-assembly behaviors of amphiphilic polymers.

To conjugate cinnamaldehyde on the side chains of polymers, three types of acid-cleavable linkages and one ROS-responsive linkage depicted in Figure 2 have been utilized. For instance, chitosan is a natural amino polysaccharide that allows the formation of imine linkages via Schiff’s base reaction between the primary amine of chitosan and the aldehyde group of cinnamaldehyde. Other polymers containing amino groups, such as gelatin, polyethyleneimine, and poly(amidoamine), can also be used to conjugate cinnamaldehyde to them via imine linkage. However, due to the branched chemical structure of polyethyleneimine and poly(amidoamine), the resulting cinnamaldehyde–polymer conjugates are also a branched structure. Although hydrazone linkage is also one of the common chemical bonds for conjugating cinnamaldehyde with polymers, it is often necessary to modify the polymer first to contain hydrazine groups. Adipic acid dihydrazide and hydrazine hydrate are the commonly used reagents for introducing hydrazine groups to polymers such as hyaluronic acid [53,54] and poly(itaconic acid) [55].

Through the formation of acetal and thioacetal linkages, cinnamaldehyde can be introduced to the side chains, the backbone of polymers, or both. For side chains, the aldehyde group of cinnamaldehyde reacts with two primary hydroxyl groups of polyalcohol (e.g., glycerol, 1,1,1-tris(hydroxymethyl)ethane, and pentaerythritol) or the sulfhydryl groups of two thiol compounds (e.g., mercaptoethanol) to form an acetal or thioacetal linkage first, and then the resulting cinnamaldehyde-derived acetal/thioacetal compounds are further grafted onto the polymers by the esterification reaction. For backbone, cinnamaldehyde typically reacts with the hydroxyl/sulfhydryl groups of two alcohol/thiol compounds and then the resulting cinnamaldehyde-derived acetal/thioacetal compounds are modified to introduce acryloyl groups as a monomer for the following polymerization via the Michael addition reaction.

**Table 2:** A Summary of Cinnamaldehyde-Conjugated Polymers with Stimuli-Responsive Linkage and Their Biomedical Applications.

Polymer	Linkage between CA and the Following Substances	Linkage	Position *	Combined Ingredients	Design Purpose	Testing Objects	Ref.
HA-CA	Hyaluronic acid	Hydrazone	S	(1) β-Phenethyl isothiocyanate (2) Protoporphyrin	Anticancer	(1) 4T1-bearing mice (2) B16F10-bearing C57BL/6 mice	[53] [54]
PIAT-CA	Poly(itaconic acid) derivative	Hydrazone	S	-	Anticancer	MCF-7 cells	[55]
Cinnamaldehyde dimer	Diethylenetriamine	Imine	-	Sorafenib; PTX	Anticancer	4T1 tumor-bearing BALB/c mice	[56]
PEG-b-PMPMC-CA	PEG-b-PMPMC	Acetal	S	(1S,3R)-RSL3	Anticancer	4T1 tumor-bearing mice	[57]
pCA; CZP	Ethanolamine; Acrylic acid 2-hydroxyethyl ester	Acetal	B	(1) DOX (2) Protoporphyrin IX zinc (II)	Anticancer	A549 tumor-bearing nude mice	[58,59]
CS-CA	Chitosan	Imine	S	(1) DOX (2) Enrofloxacin (3) Acetaminophen	(1) Anticancer (2) Treating bacterial infections (3) Excipient	(1) MCF-7/ADR tumor-bearing mice (2) *S. aureus*	[60,61,62,63,64,65,66,67,68,69,70,71]
Poly(disulfide acetal)	Hexamethylene diisocyanate; 2,2’-Dithiodiethanol	Acetal	B	DOX	Anticancer	MCF-7/ADR tumor-bearing mice	[72]
PEG-PTA_1-MT_	1,3-dimercapto-2-propanol	Thioacetal	B	1-Methyl-DL-tryptophan	Anticancer	CT26 tumor-bearing BALB/c mice	[73]
mPEG-b-poly(thioacetal-thioether)	mPEG; 2,2’-Thiodiethanol; 3-mercaptopropionic acid	Thioacetal	B	DOX	Anticancer	4T1 cells HeLa cells	[74]
POEGMA-b-PCAMA; p(Gal-b-CAMA); PEG-b-P(CAMA-co-ImOAMA)	Methacryloyl chloride; 1,1,1-Tris(hydroxymethyl)ethane	Acetal	S	(1) DOX (2) Pheophorbide A (3) ProCPT	Anticancer	(1) MCF-7/ADR cells (2) HepG2 tumor-bearing female mice (3) 4T1 tumor-bearing mice	[75] [76] [77]
mPEG5k-TA-CA-*block*-poly(TA-CA-PTX-*co*-DPA)	Methacryloyl chloride; cysteamine; mercaptoethanol	Thioacetal	B, S	PTX	Anticancer	4T1 tumor-bearing mice	[78]
Gelatin-CaCO_3_ hydrogel	Gelatin	Imine	S	-	Bone substitute	Adult male Wistar rats	[79]
PEEGE-b-PAHGE-CA	PEEGE-b-PAHGE	Imine	S	-	Anticancer	SW620 cells	[80]
Polyethyleneimine-cinnamaldehyde coating	Polyethyleneimine	Imine	-	-	Antibacterial coating	*E. coli* *S. aureus*	[81]
Dextran-CA	Dextran	Acetal	S	10-Hydroxy camptothecin	Anticancer	HCT116 tumor-bearing female mice	[82]
Pss-(NIPAm-CA-TPGS) nanogel	Dihydrazide itaconate; N-isopropylacrylamide	Hydrazone	-	-	Anticancer	MCF-7 cells	[83]
Cinnamaldehyde-conjugated maltodextrin	Maltodextrin	Acetal	S	Camptothecin -	Anticancer	SW620 tumor-bearing BALB/c mice	[84,85]
HRGP-IR	2-Hydroxyl ethyl acrylate; trimethylenedipiperidine; tyramine; mPEG acrylate	Acetal	B	Ferrocene IR-820	Nanotheranostic agent for cancer treatment	SW620 tumor-bearing nude mice	[86]
mPEG2k-b-(NTA-HD)n	3-Mercaptopropionic acid; 1,6-Hexanediol	Thioacetal	B	DOX	Anticancer	4T1 cells HeLa cells	[87]
PCAE	Acrylic acid 2-hydroxyethyl ester; trimethylene dipiperidine	Acetal	B	Camptothecin Ferrocene	(1) Anticancer; (2) Relieve coronary vasospasm; (3) Antibacterial agents	(1) SW620 tumor-bearing nude mice (2) Porcine hearts & Circumflex coronary arteries (3) Drug-resistant *P. aeruginosa*-bearing mice	[88] [89] [90]
TPE-CB-CA-TPP PUs	Pentaerythritol; Hexamethylene diisocyanate	Acetal	S	-	Anticancer	HeLa cells	[91]
Poly(amidoamine)	Poly(amidoamine)	Imine	-	Ferrocene	Anticancer	4T1 tumor-bearing BALB/c mice	[92]
P(PEG-co-(MAA-CQ))	P(PEG-coMAA)	Acetal	S	DOX	Anticancer	4T1 tumor-bearing BALB/c mice	[93]
PSO-475a	Glycerol; Methacryloyl chloride; mPEG-methacrylate	Acetal	S	Nile Red	Anticancer	A375 melanoma cells; B16 melanoma cells	[94]
TSEOP	PLG-N_3_	Acetal	S	-	Anticancer	CT26 tumor-bearing BALB/c mice 4T1 tumor-bearing BALB/c mice	[95]

* B = backbone; S = side chains; I = bridge. Abbreviations: DOX = doxorubicin; PTX = Paclitaxel; ProCPT = Phenylboronic pinacol ester-caged camptothecin; IR-820 = new indocyanine green.

In addition, cinnamaldehyde can also be utilized to link two polymer segments (e.g., PEG) as a bridge via acetal linkage [96]. Interestingly, Hirose et al. [97] reported that cinnamaldehyde can be conjugated with cellulose in an ionic liquid by an oxidative esterification reaction to synthesize cellulose cinnamate, but the release of cinnamaldehyde from cellulose cinnamate was not discussed. Manukumar et al. [98] reported that cinnamaldehyde can be grafted onto low-density polyethylene via C–O–C bond by treating them together with UV radiation at 365 nm.

## 3. The Applications in the Biomedical Field

Because cinnamaldehyde-contained polymers exhibit excellent antimicrobial activities against a variety of bacteria (e.g., Staphylococcus aureus and Escherichia coli) and fungi (e.g., Candida albicans and Botrytis cinerea), their applications have been investigated in depth in the field of food packaging over the past few decades. Cinnamaldehyde-loaded polymeric films are one of the research focuses. However, in addition to antimicrobials, cinnamaldehyde has also shown some special advantages in the biomedical field, such as promoting cellular ROS production and inhibiting MDR strains. Moreover, cinnamaldehyde can also be conveniently applied to synthesize prodrugs with other drugs (e.g., Polymyxin B [99]) and stimuli-responsive polymers for drug delivery due to its high reactivity. Therefore, this review will focus on the applications of cinnamaldehyde-contained polymers in the biomedical field.

### 3.1. The Applications of Cinnamaldehyde-Loaded Polymers

**Figure 3:**
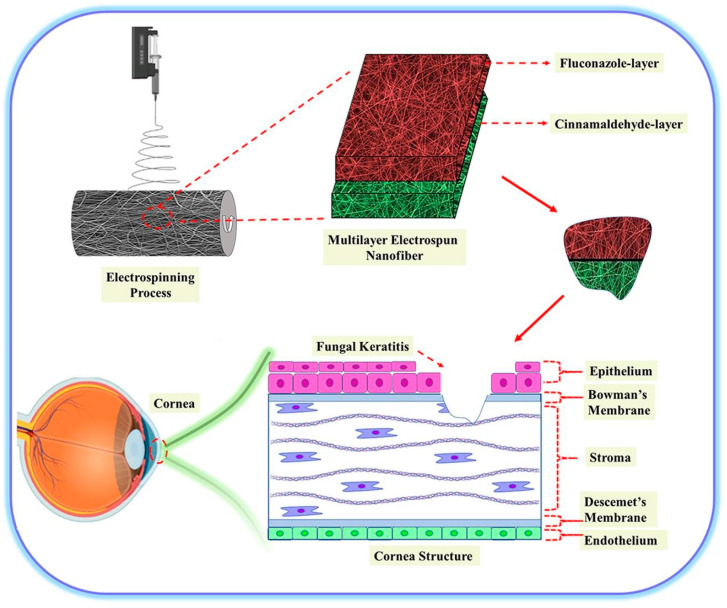
Schematic presentation of the fabrication of multilayer electrospun nanofibers for corneal tissue engineering application. Reprinted from ref. [49], Copyright (2022) with permission from Elsevier.

The antimicrobial activity of cinnamaldehyde can be used not only in the field of food packaging but also in the field of biomedicine. For example, Mishra et al. [48] prepared a cinnamaldehyde-loaded gellan/PVA nanofiber that exhibited excellent anti-biofilm activity against Candida; thus it has potential for eradicating biofilms as wound dressing material. In addition, a double-layer PVA/gelatin nanofiber loaded with cinnamaldehyde (first layer) and fluconazole (second layer) also showed that the addition of cinnamaldehyde enhanced the antifungal activity of fluconazole against *Candida albicans*, and this bilayer nanofiber may have potential to treat fungal keratitis due to its anti-biofilm activity (Figure 3) [49].

Besides antimicrobial activity, cinnamaldehyde has also been reported to be an effective ingredient that can induce apoptosis in several human tumor cells (e.g., lung cancer A549 cells, human breast cancer MCF-7 cells, and MDA-MB-231 cells) by elevating intracellular ROS levels. A nanohybrid carrier based on casein and calcium ferrite nanoparticles (casein-CFNP) was prepared by Purushothaman’s group, and cinnamaldehyde was successfully loaded into this carrier via a pH-based coacervation method. The obtained cinnamaldehyde-loaded casein-CFNP showed a controlled release of cinnamaldehyde which can be triggered by a magnetic field or acidic conditions. Significantly, the results of in vitro cell viability studies showed that the biotin-modified casein-CFNP greatly enhanced the anticancer activity against lung cancer A549 cells, and the IC50 value of cinnamaldehyde decreased from 45.89 μg/mL to 2.53 μg/mL [47]. Moreover, cinnamaldehyde can also be encapsulated into polymers along with other agents to improve therapeutic efficacy. The agents that can deplete cellular GSH or promote ROS generation are often a suitable choice to combine with cinnamaldehyde. For instance, diallyl trisulfide, as a GSH-depleting agent, had been loaded into PLGA–PEG copolymer with cinnamaldehyde together to prepare nanoparticles for enhancing the effect of tumor suppression [46].

In addition, cinnamaldehyde can also be reacted with other agents to obtain prodrugs first, and then the resulting cinnamaldehyde prodrugs are loaded into polymeric nanoparticles [100]. For example, the prodrug based on two dopamine molecules and one cinnamaldehyde molecule was synthesized via thioacetal linkage and then loaded into P-SS-D (an amphiphilic polymer)-based polymeric nanoparticles with Fe^3+^/Gd^3+^ by the nanoprecipitation method. The cinnamaldehyde prodrug-loaded nanoparticles exhibited great potential for magnetic resonance imaging-based visual tumor treatment [101]. Moreover, ferrocene is an iron-containing catalyst to enhance chemodynamic therapy efficacy by promoting the Fenton reaction, and has host–guest interaction with the hydrophobic cavity of β-cyclodextrin. Thus, Xu et al. designed a ferrocene-modified cinnamaldehyde prodrug with a hydrazone linkage, and then loaded this prodrug to β-cyclodextrin-functionalized hyaluronic acid (HA-CD) via host–guest interactions between the ferrocene moieties in the prodrug and β-cyclodextrin moieties of HA-CD. The combination of activating tumor-specific oxidative stress amplification and cascading enhancement of the Fenton reaction results in a superior cancer therapeutic effect [102].

Although cinnamaldehyde has a variety of biological activities, treatment with cinnamaldehyde alone has limited efficacy. As can be seen from the data in Table 1, loading cinnamaldehyde into the polymers to achieve its controlled or sustained release can usually prolong its treatment time, but it is difficult to greatly enhance its therapeutic effect [40,48]. This may limit the biomedical applications of cinnamaldehyde-loaded polymers. Surprisingly, the combination of cinnamaldehyde with other active ingredients/drugs (e.g., DATS [46] and biotin [47]) often shows excellent synergistic effects. Therefore, for cinnamaldehyde-loaded polymers, studying the synergy between cinnamaldehyde and other active ingredients/drugs may be beneficial to improve their therapeutic effects.

In addition, the treatment effects of cinnamaldehyde-loaded polymers are also affected by the dosage form and the biological activity of the polymers themselves (e.g., chitosan has bacteriostatic and anti-inflammatory properties). Therefore, multifunctional cinnamaldehyde carriers may be a potential research direction.

### 3.2. The Applications of Cinnamaldehyde-Conjugated Polymers

Unlike cinnamaldehyde-loaded polymers, cinnamaldehyde-conjugated polymers not only enable controlled and/or sustained release of cinnamaldehyde but also can be used as drug delivery systems. Thus, cinnamaldehyde-conjugated polymers have been extensively studied for various biomedical purposes, such as pharmaceutical excipients, wound dressings, anti-inflammation, and anti-tumor.

#### 3.2.1. Controlled or Sustained Release of Cinnamaldehyde

For controlled or sustained release, cinnamaldehyde is commonly introduced to the side chains of polymers via acid-cleavable linkages to obtain cinnamaldehyde-conjugated polymers with pH responsiveness. As mentioned in Section 2.2, chitosan is one of the most studied polymers in cinnamaldehyde-conjugated polymers due to the amino groups in its structural units. Chitosan–cinnamaldehyde conjugates, denoted as CS–CA, exhibit higher antibacterial and antioxidative effects than chitosan due to the acid-cleavable imine linkages between chitosan and cinnamaldehyde moieties; thus it may be applied as an excipient to replace chitosan in pharmaceutical formulations (e.g., tablet) for delivering active pharmaceutical ingredients such as acetaminophen [70].

Gelatin is a kind of protein and also has many functional groups, including amino groups. Dewi et al. prepared a Plaster of Paris–CaCO_3_ hydrogel containing gelatin, in which gelatin was conjugated with cinnamaldehyde via imine linkages, and the results regarding physical properties in vivo biocompatibility showed that it may be a promising bone substitute containing cinnamaldehyde as an anti-inflammatory agent [79].

**Figure 4:**
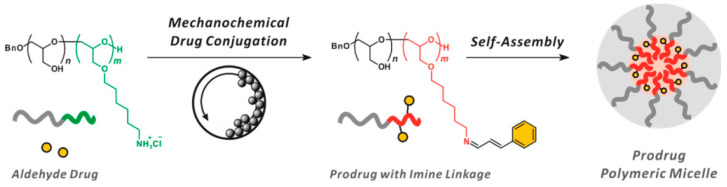
Schematic representation of the prodrug approach via mechanochemical drug conjugation via pH-responsive imine linkage. Reprinted (adapted) with permission from ref. [80]. Copyright 2021 American Chemical Society.

Moreover, cinnamaldehyde can also be released as an oxidative stress-inducing chemotherapeutic agent from cinnamaldehyde-conjugated polymers for the treatment of cancer. For instance, cinnamaldehyde was conjugated with diblock copolymer PEEGE-b-PAHGE via imine linkage, and the resulting conjugate can self-assemble to form polymeric micelles that can release cinnamaldehyde to induce colon cancer SW620 cells’ apoptosis with the disintegration of micelles under acidic conditions by cleaving the imine bonds (Figure 4) [80]. In addition, the controlled release of cinnamaldehyde can also be combined with other strategies (e.g., near-infrared laser-induced photothermal therapy [85]) to achieve better anticancer efficacy.

#### 3.2.2. Drug Delivery

**Figure 5:**
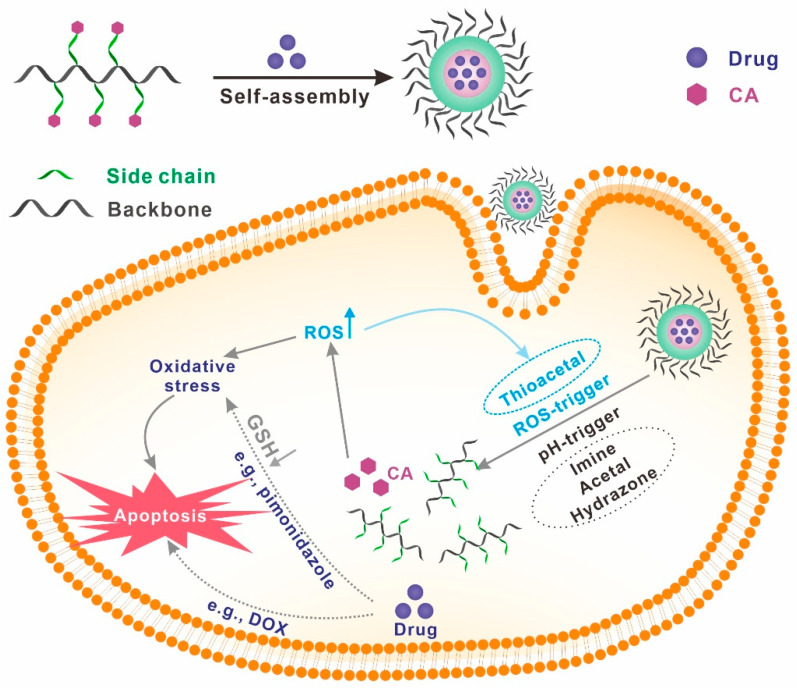
Schematic diagram of the typical anticancer mechanism of stimuli-responsive polymers containing cinnamaldehyde moieties.

For drug delivery, cinnamaldehyde–polymer conjugates can also be used, in which cinnamaldehyde can be present in their side chains and/or backbone. The simplest strategy for preparing cinnamaldehyde–polymer conjugates for drug delivery is to graft cinnamaldehyde directly to the side chains of the polymers through cleavable linkage. Thus, the resulting cinnamaldehyde–polymer conjugates are generally responsive to external stimuli (e.g., pH and ROS), enabling the controlled release of encapsulated drugs. In addition, the released cinnamaldehyde can elevate intracellular ROS level and further accelerate the degradation of ROS-responsive polymers (thioacetal linkage), while the released drugs can directly kill cancer cells or accelerate the apoptosis of cancer cells by consuming GSH to enhance oxidative stress (Figure 5).

##### Polymers with Cinnamaldehyde in the Side Chains

Polysaccharides are commonly used to prepare conjugated polymers containing cinnamaldehyde in the side chains due to their excellent biocompatibility and biodegradability. For instance, Liu et al. synthesized a pH-responsive cinnamaldehyde–hyaluronic acid conjugate containing hydrazone linkage, which can be applied to deliver drugs (e.g., β-phenethyl isothi-ocyanate) for tumor treatment [53,54]. In addition, Yang et al. conjugated cinnamaldehyde with chitosan via imine linkages and the obtained CS–CA conjugates were used as a drug carrier to load the broad-spectrum antibacterial agent enrofloxacin. Enrofloxacin was released faster in an acidic environment (pH 5.0) than in a normal environment (pH 7.4). It may be used to target the treatment of sites infected by acid-producing bacteria [63]. Besides hyaluronic acid and chitosan, cinnamaldehyde has also been conjugated with dextran via acetal linkages. The resulting dextran–CA conjugate can also self-assemble into nanoparticles in an aqueous solution, and 10-hydroxy camptothecin has been successfully encapsulated into the self-assembled nanoparticles as a model drug. Both 10-hydroxy camptothecin and cinnamaldehyde can be fast-released from dextran–CA conjugate in an acidic condition via the cleavage of acetal linkages and have shown good synergistic anticancer effects against colon cancer HCT116 cells in both in vitro and in vivo anticancer studies [82]. Moreover, a starch glycolate and cinnamaldehyde conjugate with acetal or hemiacetal linkage has also been reported, and it can be applied as a gastro retentive drug delivery system to prepare an artesunate emulsion for the treatment of *H. pylori* infection [103].

In comparison with single pH-responsive polymers, dual and multi-responsive polymers are more promising as drug carriers, which can both improve therapeutic efficacy and reduce side effects [104]. For instance, Chen et al. designed a novel dual pH-responsive chitosan derivative, DCCA, containing both β-carboxylic amide and imine bonds, corresponding to responsive pH values of 6.5 (tumor extracellular pH) and 5.0 (intracellular pH), respectively. Doxorubicin was successfully loaded in the nanoparticles prepared from DCCA. In the tumor extracellular environment, the surface charge of DOX-loaded DCCA nanoparticles reversed from negative (−6.3 mV, -COOH) to positive (+11.4 mV, -NH_2_) due to the break of β-carboxylic amide, which improved cellular uptake efficiency. When DOX-loaded DCCA nanoparticles were taken up by the tumor cells, the cleavage of imine linkages under intracellular pH trigged the release of cinnamaldehyde and disrupted the hydrophilic/hydrophobic balance of DCCA, ultimately releasing DOX. In vivo anticancer results showed that the DOX-loaded DCCA nanoparticles not only could induce more tumor cell apoptosis, with an inhibition rate of up to 84.94%, but also decrease the adverse effects of DOX [62].

##### Polymers with Cinnamaldehyde in the Backbone

Cleavage of the polymer backbone often results in different stimuli-responsive behaviors of the polymer (e.g., faster response speed) due to the loss of structural integrity. Xu’s group designed amphiphilic copolymers poly(thioacetal-thioether) and poly(ester-thioacetal), in which cinnamaldehyde binds to other structural units on the backbone via ROS-responsive thioacetal linkages. DOX was encapsulated into poly(thioacetal-thioether)/poly(ester-thioacetal)-based micelles. The obtained DOX-loaded micelles can respond to the high concentration of ROS in tumor cells via the cleavage of thioacetal linkages, resulting in the degradation of poly(thioacetal-thioether)/poly(ester-thioacetal) to rapidly release DOX and cinnamaldehyde. The released cinnamaldehyde can further promote the generation of ROS, forming a synergistic effect with DOX to accelerate the apoptosis of tumor cells [74,87].

Additionally, Raffai et al. prepared an amphiphilic polymer containing cinnamaldehyde in its backbone with acetal linkages (PCAE), and found that its micelles had vasodilator properties different from the relaxation mechanism of cinnamaldehyde. Thus, it may be applied to relieve coronary vasospasm [89]. Moreover, PCAE also has been reported to load ferrous ions successfully. In vivo results of mice revealed that the ferrocene-loaded PCAE micelles can reduce pulmonary infection and lung damage [90].

##### Polymers with Cinnamaldehyde in Both Side Chains and Backbone

So far, only a limited number of polymers that contain cinnamaldehyde in both side chains and backbone have been reported. Wang et al. designed a novel amphiphilic block copolymer mPEG5k-TA-CA-block-poly(TA-CA-PTX-co-DPA), in which cinnamaldehyde moieties were incorporated in both side chains and backbone via thioacetal linkage [78]. In addition, paclitaxel (PTX) was also conjugated in the side chains of mPEG5k-TA-CA-block-poly(TA-CA-PTX-co-DPA). This amphiphilic block copolymer can self-assemble into micelles which release the conjugated PTX and cinnamaldehyde via the cleavage of ROS-responsive thioacetal linkages after endocytosis into cancer cells. Then, the release of PTX was further enhanced owing to the generation of ROS that was promoted by the released cinnamaldehyde, thus accelerating the apoptosis of cancer cells. This cascaded ROS-feedback strategy may be an effective way to develop cinnamaldehyde-conjugated polymers for cancer treatment.

Compared to cinnamaldehyde-loaded polymers, cinnamaldehyde-conjugated polymers have higher designability and generally exhibit better treatment effects. As can be seen from Table 2, some cinnamaldehyde-conjugated polymers consist of non-degradable carbon–carbon backbone, which may limit their biomedical applications due to their poor degradability. Cinnamaldehyde-conjugated polymers containing cinnamaldehyde moieties in their backbone generally have good biodegradability due to the presence of cleavage linkages in their backbone, but the degradation products need to be considered due to their irritation, toxicity, and safety. Therefore, designing biodegradable, non-toxic and safer cinnamaldehyde-conjugated polymers may be the future development trend.

## 4. Conclusions

Cinnamaldehyde is a natural product that exhibits various biological activities including antimicrobial, anti-inflammatory, and anticancer, but the application of cinnamaldehyde is limited by its sensitivity to light and poor water solubility. In order to obtain long-lasting, better treatment effects and extend its applications, cinnamaldehyde can be loaded into polymers for sustained and/or controlled release. Moreover, cinnamaldehyde can also be modified to form a prodrug before being loaded into polymers, or directly loaded into polymers with other agents to obtain a synergistic therapeutic effect. Therefore, although many cinnamaldehyde-loaded polymers are investigated in the food field, they also show good application prospects in the biomedical field.

Additionally, cinnamaldehyde can also be conjugated in the side chains or the backbone via cleavable linkages for preparing stimuli-responsive cinnamaldehyde-conjugated polymers as smart drug delivery systems. The stimuli-responsive behavior of cinnamaldehyde-conjugated polymers not only induces the release of cinnamaldehyde but also triggers the release of encapsulated drugs due to the degradation of polymers. On the other hand, as a ROS generation agent, the released cinnamaldehyde can enhance intracellular ROS levels for amplified oxidative stress. Therefore, cinnamaldehyde-conjugated polymers exhibit great potential for biomedical applications, especially for cancer treatment.

In conclusion, due to the biological activities of cinnamaldehyde and its convenience for the design and preparation of stimuli-responsive polymers, cinnamaldehyde-contained polymers show tremendous promise in the biomedical field. It is expected that more interesting cinnamaldehyde-contained polymers, such as cinnamaldehyde-contained poly(amino acid)s derivatives, will be designed for biomedical applications in the future. In view of the fact that poly(amino acid)s have attracted much attention in the field of biomedicine, and their easily functionalized and biodegradable properties, cinnamaldehyde-contained poly(amino acid)s derivatives may be a promising direction. In addition, the synergy between cinnamaldehyde and other drugs or ingredients is also worth paying attention to and studying in depth.

## Data Availability

Not applicable.

## References

[1] Adetunji C.O., Palai S., Ekwuabu C.P., Egbuna C., Adetunji J.B., Ehis-Eriakha C.B., Kesh S.S., Mtewa A.G., Egbuna C., Mishra A.P., Goyal M.R. (2021). Chapter 1—General principle of primary and secondary plant metabolites: Biogenesis, metabolism, and extraction. Preparation of Phytopharmaceuticals for the Management of Disorders.

[2] Calo J.R., Crandall P.G., O’Bryan C.A., Ricke S.C. (2015). Essential oils as antimicrobials in food systems—A review. Food Control.

[3] Stevens N., Allred K. (2022). Antidiabetic Potential of Volatile Cinnamon Oil: A Review and Exploration of Mechanisms Using In Silico Molecular Docking Simulations. Molecules.

[4] de Souza A.G., da Silva Barbosa R.F., Quispe Y.M., Rosa D.D.S. (2022). Essential oil microencapsulation with biodegradable polymer for food packaging application. J. Polym. Environ..

[5] Maes C., Abir S., Jacquet P., De Clerck C., Blecker C., Bouquillon S., Fauconnier M.-L. (2022). Cinnamomum zeylanicum Essential Oil Formulation with Poly(propylene imine) Dendrimers with Surface-Grafted Glycerol: Release Kinetics of trans-Cinnamaldehyde and Germination Inhibition Effects. J. Agric. Food Chem..

[6] Ahmad-Mudzaqqir M.Y., Wong Y.C., Wan-Nurdiyana W.A. (2014). Extraction of Essential Oil from Cinnamon (*Cinnamomum zeylanicum*). Orient. J. Chem..

[7] Zaio Y.P., Gatti G., Ponce A.A., Larralde N.A.S., Martinez M.J., Zunino M.P., Zygadlo J.A. (2018). Cinnamaldehyde and related phenylpropanoids, natural repellents, and insecticides against *Sitophilus zeamais* (Motsch.). A chemical structure-bioactivity relationship. J. Sci. Food Agric..

[8] Thirapanmethee K., Kanathum P., Khuntayaporn P., Huayhongthong S., Surassmo S., Chomnawang M.T. (2021). Cinnamaldehyde: A plant-derived antimicrobial for overcoming multidrug-resistant Acinetobacter baumannii infection. Eur. J. Integr. Med..

[9] Chun J.Y., Jo Y.J., Bjrapha P., Choi M.J., Min S.G. (2015). Antimicrobial Effect of alpha- or beta-Cyclodextrin Complexes with Trans-Cinnamaldehyde Against *Staphylococcus aureus* and *Escherichia coli*. Dry. Technol..

[10] Wei Q.Y., Xiong J.J., Jiang H., Zhang C., Ye W. (2011). The antimicrobial activities of the cinnamaldehyde adducts with amino acids. Int. J. Food Microbiol..

[11] Zhu Y.J., Song K.K., Li Z.C., Pan Z.Z., Guo Y.J., Zhou J.J., Wang Q., Liu B., Chen Q.X. (2009). Antityrosinase and Antimicrobial Activities of trans-Cinnamaldehyde Thiosemicarbazone. J. Agric. Food Chem..

[12] Bi Z.J., Fang S.M., Gao Q., Lei Y.F., Morrell J.J., Yan L. (2022). Improvement of mould resistance of wood with cinnamaldehyde chitosan emulsion. Wood Sci. Technol..

[13] Qureshi K.A., Mohammed S.A.A., Khan O., Ali H.M., El-Readi M.Z., Mohammed H.A. (2022). Cinnamaldehyde-Based Self-Nanoemulsion (CA-SNEDDS) Accelerates Wound Healing and Exerts Antimicrobial, Antioxidant, and Anti-Inflammatory Effects in Rats&rsquo; Skin Burn Model. Molecules.

[14] Safaei F., Tamaddonfard E., Imani M., Nafisi S. (2021). Effects of intraperitoneal and intracerebroventricular injection of cinnamaldehyde and yohimbine on blood glucose and serum insulin concentrations in ketamine-xylazine induced acute hyperglycemia. Vet. Res. Forum.

[15] Al Tbakhi B., Nsairat H., Alshaer W., Al-Kadash A., Helal W., Alrawashdeh L., Day A., Assaf K.I., Hassouneh R., Odeh F. (2022). Cinnamaldehyde–cucurbituril complex: Investigation of loading efficiency and its role in enhancing cinnamaldehyde in vitro anti-tumor activity. RSC Adv..

[16] Hong S.-H., Ismail I.A., Kang S.-M., Han D.C., Kwon B.-M. (2016). Cinnamaldehydes in Cancer Chemotherapy. Phytother. Res..

[17] Yu J.-N., Yue C.-F., Wang K.-J., Chi N.-N., Li X. (2020). Effect of cinnamaldehyde on Cav-1 and Survivin expression in epilepsy A protocol of systematic review and meta-analysis. Medicine.

[18] Friedman M. (2017). Chemistry, Antimicrobial Mechanisms, and Antibiotic Activities of Cinnamaldehyde against Pathogenic Bacteria in Animal Feeds and Human Foods. J. Agric. Food Chem..

[19] Ka H., Park H.-J., Jung H.-J., Choi J.-W., Cho K.-S., Ha J., Lee K.-T. (2003). Cinnamaldehyde induces apoptosis by ROS-mediated mitochondrial permeability transition in human promyelocytic leukemia HL-60 cells. Cancer Lett..

[20] da Nóbrega Alves D., Melo A.K.V., Alves A.F., de Araújo M.R.C., da Silva Araújo R., de Castro R.D. (2022). Safety and tolerability of cinnamaldehyde in orabase for oral candidiasis treatment: Phase I clinical trial. Clin. Oral Investig..

[21] Molania T., Malekzadeh Shafaroudi A., Saeedi M., Moosazadeh M., Valipour F., Rostamkalaei S.S., Salehabadi N., Salehi M. (2022). Evaluation of cinnamaldehyde mucoadhesive patches on minor recurrent aphthous stomatitis: A randomized, double-blind, placebo-controlled clinical trial. BMC Oral Health.

[22] Dizdarevic A., Maric M., Shahzadi I., Efiana N.A., Matuszczak B., Bernkop-Schnurch A. (2021). Imine bond formation as a tool for incorporation of amikacin in self-emulsifying drug delivery systems (SEDDS). Eur. J. Pharm. Biopharm..

[23] Cionti C., Taroni T., Sabatini V., Meroni D. (2021). Nanostructured Oxide-Based Systems for the pH-Triggered Release of Cinnamaldehyde. Materials.

[24] Zhang X.B., Ma H.Y., Sun T.D., Lei P., Yang X.L., Zhang X.M., Ling Y. (2019). Design, Synthesis and Fungicidal Activity of Novel Piperidine Containing Cinnamaldehyde Thiosemicarbazide Derivatives. Chin. J. Org. Chem..

[25] Wang Z., Yao J., Guan Z., Wu H., Cheng H., Yan G., Tang R. (2021). pH-triggered small molecule nano-prodrugs emulsified from tryptamine-cinnamaldehyde twin drug for targeted synergistic glioma therapy. Colloids Surf. B Biointerfaces.

[26] Qin F., Zhou H., Li J., Liu J., Wang Y., Bai R., Liu S., Ma M., Liu T., Gao F. (2021). Hypoxia and pH co-triggered oxidative stress amplifier for tumor therapy. Eur. J. Pharmacol..

[27] Shreaz S., Wani W.A., Behbehani J.M., Raja V., Irshad M., Karched M., Ali I., Siddiqi W.A., Hun L.T. (2016). Cinnamaldehyde and its derivatives, a novel class of antifungal agents. Fitoterapia.

[28] Doyle A.A., Stephens J.C. (2019). A review of cinnamaldehyde and its derivatives as antibacterial agents. Fitoterapia.

[29] Chen B.-J., Fu C.-S., Li G.-H., Wang X.-N., Lou H.-X., Ren D.-M., Shen T. (2017). Cinnamaldehyde Analogues as Potential Therapeutic Agents. Mini-Rev. Med. Chem..

[30] Hosseini S.F., Kaveh F., Schmid M. (2022). Facile fabrication of transparent high-barrier poly(lactic acid)-based bilayer films with antioxidant/antimicrobial performances. Food Chem..

[31] Qin Y.Y., Yang J.Y., Xue J. (2015). Characterization of antimicrobial poly(lactic acid)/poly(trimethylene carbonate) films with cinnamaldehyde. J. Mater. Sci..

[32] Akgün M., Başaran İ., Suner S.C., Oral A. (2020). Geraniol and cinnamaldehyde as natural antibacterial additives for poly(lactic acid) and their plasticizing effects. J. Polym. Eng..

[33] Zhang L., Huang C., Xu Y., Huang H., Zhao H., Wang J., Wang S. (2020). Synthesis and characterization of antibacterial polylactic acid film incorporated with cinnamaldehyde inclusions for fruit packaging. Int. J. Biol. Macromol..

[34] Siddiqui M.N., Redhwi H.H., Tsagkalias I., Vouvoudi E.C., Achilias D.S. (2021). Development of Bio-Composites with Enhanced Antioxidant Activity Based on Poly(lactic acid) with Thymol, Carvacrol, Limonene, or Cinnamaldehyde for Active Food Packaging. Polymers.

[35] Ge X., Huang X., Zhou L., Wang Y. (2022). Essential oil-loaded antimicrobial and antioxidant zein/poly(lactic acid) film as active food packaging. Food Packag. Shelf Life.

[36] Srisa A., Harnkarnsujarit N. (2020). Antifungal films from trans-cinnamaldehyde incorporated poly(lactic acid) and poly(butylene adipate-co-terephthalate) for bread packaging. Food Chem..

[37] Muller J., González-Martínez C., Chiralt A. (2017). Poly(lactic) acid (PLA) and starch bilayer films, containing cinnamaldehyde, obtained by compression moulding. Eur. Polym. J..

[38] Kardam S.K., Kadam A.A., Dutt D. (2021). Retention of cinnamaldehyde in poly(vinyl alcohol) films intended for preservation of faba beans through vapor-phase antimicrobial effect. Food Packag. Shelf Life.

[39] Chen C., Zong L., Wang J., Xie J. (2021). Microfibrillated cellulose reinforced starch/polyvinyl alcohol antimicrobial active films with controlled release behavior of cinnamaldehyde. Carbohydr. Polym..

[40] Canales D., Montoille L., Rivas L.M., Ortiz J.A., Yanez S.M., Rabagliati F.M., Ulloa M.T., Alvarez E., Zapata P.A. (2019). Fungicides Films of Low-Density Polyethylene (LDPE)/Inclusion Complexes (Carvacrol and Cinnamaldehyde) Against Botrytis Cinerea. Coatings.

[41] Yeldir E.K., Oral A. (2021). Environmentally-friendly preparation of chitosan microspheres and encapsulation studies of cinnamaldehyde: A convenient sustained release system for cinnamaldehyde. Maced. J. Chem. Chem. Eng..

[42] Subhaswaraj P., Barik S., Macha C., Chiranjeevi P.V., Siddhardha B. (2018). Anti quorum sensing and anti biofilm efficacy of cinnamaldehyde encapsulated chitosan nanoparticles against *Pseudomonas aeruginosa* PAO1. LWT.

[43] Joghataei M., Hosseini S.F., Arab-Tehrany E. (2019). Cinnamaldehyde loaded chitosan/tripolyphosphate nanoassemblies: Fabrication, characterization, and in vitro evaluation of antioxidant activity. J. Food Process. Preserv..

[44] Wang X.W., Cheng F.Y., Wang X.J., Feng T.T., Xia S.Q., Zhang X.M. (2021). Chitosan decoration improves the rapid and long-term antibacterial activities of cinnamaldehyde-loaded liposomes. Int. J. Biol. Macromol..

[45] Gursu B.Y., Dag I., Dikmen G. (2022). Antifungal and antibiofilm efficacy of cinnamaldehyde-loaded poly(DL-lactide-co-glycolide) (PLGA) nanoparticles against *Candida albicans*. Int. Microbiol..

[46] Liu Y., Liu H., Wang L., Wang Y., Zhang C., Wang C., Yan Y., Fan J., Xu G., Zhang Q. (2020). Amplification of oxidative stress via intracellular ROS production and antioxidant consumption by two natural drug-encapsulated nanoagents for efficient anticancer therapy. Nanoscale Adv..

[47] Purushothaman B.K., Maheswari P.U., Sheriffa Begum K.M.M. (2021). pH and magnetic field responsive protein-inorganic nanohybrid conjugated with biotin: A biocompatible carrier system targeting lung cancer cells. J. Appl. Polym. Sci..

[48] Mishra P., Gupta P., Pruthi V. (2021). Cinnamaldehyde incorporated gellan/PVA electrospun nanofibers for eradicating *Candida biofilm*. Mater. Sci. Eng. C.

[49] Ilhan E., Cesur S., Sulutas R.B., Pilavci E., Dalbayrak B., Kaya E., Arisan E.D., Tinaz G.B., Sengor M., Kijeńska-Gawrońska E. (2022). The Role of Multilayer Electrospun Poly(Vinyl Alcohol)/Gelatin nanofibers loaded with Fluconazole and Cinnamaldehyde in the Potential Treatment of Fungal Keratitis. Eur. Polym. J..

[50] Shi Y., Shen J., Yang D., Du J., Lu S., Ma M., He H., Chen S., Wang X. (2021). Green cinnamaldehyde and thymol modified zinc oxide with double synergistic antibacterial effects in polypropylene. J. Appl. Polym. Sci..

[51] Qian Z.-J., Zhang J., Xu W.-R., Zhang Y.-C. (2022). Development of active packaging films based on liquefied shrimp shell chitin and polyvinyl alcohol containing β-cyclodextrin/cinnamaldehyde inclusion. Int. J. Biol. Macromol..

[52] Yildiz Z.I., Kilic M.E., Durgun E., Uyar T. (2019). Molecular Encapsulation of Cinnamaldehyde within Cyclodextrin Inclusion Complex Electrospun Nanofibers: Fast-Dissolution, Enhanced Water Solubility, High Temperature Stability, and Antibacterial Activity of Cinnamaldehyde. J. Agric. Food Chem..

[53] Liu Q., Ding X., Xu X., Lai H., Zeng Z., Shan T., Zhang T., Chen M., Huang Y., Huang Z. (2022). Tumor-targeted hyaluronic acid-based oxidative stress nanoamplifier with ROS generation and GSH depletion for antitumor therapy. Int. J. Biol. Macromol..

[54] Xu X., Huang B., Zeng Z., Chen J., Huang Z., Guan Z., Chen M., Huang Y., Zhao C. (2020). Broaden sources and reduce expenditure: Tumor-specific transformable oxidative stress nanoamplifier enabling economized photodynamic therapy for reinforced oxidation therapy. Theranostics.

[55] Dong K., Lei Q., Qi H., Zhang Y., Cui N., Wu X., Xie L., Yan X., Lu T. (2019). Amplification of Oxidative Stress in MCF-7 Cells by a Novel pH-Responsive Amphiphilic Micellar System Enhances Anticancer Therapy. Mol. Pharm..

[56] Zhou Z., Liang H., Yang R., Yang Y., Dong J., Di Y., Sun M. (2022). Glutathione Depletion-Induced Activation of Dimersomes for Potentiating the Ferroptosis and Immunotherapy of “Cold” Tumor. Angew. Chem. Int. Ed..

[57] Yan Z., Wu S., Zhou Y., Li F. (2022). Acid-Responsive Micelles Releasing Cinnamaldehyde Enhance RSL3-Induced Ferroptosis in Tumor Cells. ACS Biomater. Sci. Eng..

[58] Lee Y., Song N., Kim N., Yang M., Kwon G., Hyeon H., Jung E., Park S.-C., Kim C., Lee D. (2022). Oxidative Stress Amplifying Polyprodrug Micelles as Drug Carriers for Combination Anticancer Therapy. Biomacromolecules.

[59] Noh J., Jung E., Lee J., Hyun H., Hong S., Lee D. (2019). Engineered Polymeric Micelles for Combinational Oxidation Anticancer Therapy through Concurrent HO-1 Inhibition and ROS Generation. Biomacromolecules.

[60] Gadkari R.R., Gupta A., Teke U., Awadhiya A., Shahadat M., Ali W., Das A., Alagirusamy R. (2021). A sustainable way for surface functionalisation of PET nonwoven with novel chitosan-cinnamaldehyde cross-linked nanoparticles. J. Ind. Eng. Chem..

[61] Heras-Mozos R., Hernández R., Gavara R., Hernández-Muñoz P. (2022). Dynamic covalent chemistry of imines for the development of stimuli-responsive chitosan films as carriers of sustainable antifungal volatiles. Food Hydrocoll..

[62] Chen Q., Jia C., Xu Y., Jiang Z., Hu T., Li C., Cheng X. (2022). Dual-pH responsive chitosan nanoparticles for improving in vivo drugs delivery and chemoresistance in breast cancer. Carbohydr. Polym..

[63] Yang J.-L., Yuan H.-Q., Liu B.-S., He J.-X., Fan Q., Deng K., Song D., Bao G.-M. (2021). Facile one-pot synthesis of chitosan-based nanoparticles for pH-responsive enrofloxacin delivery. Mater. Today Commun..

[64] Liu L., Zhu L., Zhang S., Ma Y., Wang L., Wang H., Niu X. (2021). Preparation and properties of chitosan-based bacteriostatic agents and their application in strawberry bacteriostatic preservation. J. Food Sci..

[65] Arnon-Rips H., Cohen Y., Saidi L., Porat R., Poverenov E. (2021). Covalent linkage of bioactive volatiles to a polysaccharide support as a potential approach for preparing active edible coatings and delivery systems for food products. Food Chem..

[66] Chugh B., Singh A.K., Poddar D., Thakur S., Pani B., Jain P. (2020). Relation of degree of substitution and metal protecting ability of cinnamaldehyde modified chitosan. Carbohydr. Polym..

[67] Chen H., Zhao R., Hu J., Wei Z., McClements D.J., Liu S., Li B., Li Y. (2020). One-Step Dynamic Imine Chemistry for Preparation of Chitosan-Stabilized Emulsions Using a Natural Aldehyde: Acid Trigger Mechanism and Regulation and Gastric Delivery. J. Agric. Food Chem..

[68] Gadkari R.R., Suwalka S., Yogi M.R., Ali W., Das A., Alagirusamy R. (2019). Green synthesis of chitosan-cinnamaldehyde cross-linked nanoparticles: Characterization and antibacterial activity. Carbohydr. Polym..

[69] Marin L., Moraru S., Popescu M.-C., Nicolescu A., Zgardan C., Simionescu B.C., Barboiu M. (2014). Out-of-Water Constitutional Self-Organization of Chitosan–Cinnamaldehyde Dynagels. Chem. A Eur. J..

[70] Ren G., Clancy C., Tamer T.M., Schaller B., Walker G.M., Collins M.N. (2019). Cinnamyl O-amine functionalized chitosan as a new excipient in direct compressed tablets with improved drug delivery. Int. J. Biol. Macromol..

[71] Tamer T.M., Hassan M.A., Omer A.M., Valachová K., Eldin M.S.M., Collins M.N., Šoltés L. (2017). Antibacterial and antioxidative activity of O-amine functionalized chitosan. Carbohydr. Polym..

[72] Zong Q., Wang K., Xiao X., Jiang M., Li J., Yuan Y., Wang J. (2021). Amplification of tumor oxidative stresses by Poly(disulfide acetal) for multidrug resistance reversal. Biomaterials.

[73] Tu Y., Xiao X., Dong Y., Li J., Liu Y., Zong Q., Yuan Y. (2022). Cinnamaldehyde-based poly(thioacetal): A ROS-awakened self-amplifying degradable polymer for enhanced cancer immunotherapy. Biomaterials.

[74] Xu C., Han R., Liu H., Zhu Y., Zhang J., Xu L. (2021). Construction of Polymeric Micelles for Improving Cancer Chemotherapy by Promoting the Production of Intracellular Reactive Oxygen Species and Self-Accelerating Drug Release. ChemistrySelect.

[75] Xi L., Wang J., Wang Y., Ge Z. (2021). Dual-Targeting Polymeric Nanocarriers to Deliver ROS-Responsive Prodrugs and Combat Multidrug Resistance of Cancer Cells. Macromol. Biosci..

[76] Feng Z., Guo J., Liu X., Song H., Zhang C., Huang P., Dong A., Kong D., Wang W. (2020). Cascade of reactive oxygen species generation by polyprodrug for combinational photodynamic therapy. Biomaterials.

[77] Lu N., Xi L., Zha Z., Wang Y., Han X., Ge Z. (2021). Acid-responsive endosomolytic polymeric nanoparticles with amplification of intracellular oxidative stress for prodrug delivery and activation. Biomater. Sci..

[78] Wang B., Chen K., Zhang Q., Gu L., Luo Q., Li Z., Gong Q., Zhang H., Gu Z., Luo K. (2021). ROS-responsive amphiphilic block copolymer-drug conjugate: Design, synthesis and potential as an efficient drug delivery system via a positive feedback strategy. Chem. Eng. J..

[79] Dewi A.H., Yulianto D.K., Siswomihardjo W., Rochmadi R., Ana I.D. (2021). Effect of Dehydrothermal Treatment on the Mechanical Properties and Biocompatibility of Plaster of Paris-CaCO3 Hydrogel Loaded With Cinnamaldehyde for Biomedical Purposes. Nat. Prod. Commun..

[80] Han S., Lee J., Jung E., Park S., Sagawa A., Shibasaki Y., Lee D., Kim B.-S. (2021). Mechanochemical Drug Conjugation via pH-Responsive Imine Linkage for Polyether Prodrug Micelles. ACS Appl. Bio Mater..

[81] Cox H.J., Li J., Saini P., Paterson J.R., Sharples G.J., Badyal J.P.S. (2021). Bioinspired and eco-friendly high efficacy cinnamaldehyde antibacterial surfaces. J. Mater. Chem. B.

[82] Zhao C.W., Cao W.L., Zheng H.L., Xiao Z.X., Hu J., Yang L.H., Chen M., Liang G., Zheng S.Q., Zhao C.G. (2019). Acid-responsive nanoparticles as a novel oxidative stress-inducing anticancer therapeutic agent for colon cancer. Int. J. Nanomed..

[83] Dong K., Lei Q.Y., Guo R.H., Wu X.L., Zhang Y.N., Cui N., Shi J.Y., Lu T.L. (2019). Regulating intracellular ROS signal by a dual pH/reducing-responsive nanogels system promotes tumor cell apoptosis. Int. J. Nanomed..

[84] Yoo W., Yoo D., Hong E., Jung E., Go Y., Singh S.V.B., Khang G., Lee D. (2018). Acid-activatable oxidative stress-inducing polysaccharide nanoparticles for anticancer therapy. J. Control. Release.

[85] Hong E., Hyun H., Lee H., Jung E., Lee D. (2020). Acid-sensitive oxidative stress inducing and photoabsorbing polysaccharide nanoparticles for combinational anticancer therapy. Int. J. Pharm..

[86] Yang W., Noh J., Park H., Gwon S., Singh B., Song C., Lee D. (2018). Near infrared dye-conjugated oxidative stress amplifying polymer micelles for dual imaging and synergistic anticancer phototherapy. Biomaterials.

[87] Xu L., Zhao M., Zhang H., Gao W., Guo Z., Zhang X., Zhang J., Cao J., Pu Y., He B. (2018). Cinnamaldehyde-Based Poly(ester-thioacetal) To Generate Reactive Oxygen Species for Fabricating Reactive Oxygen Species-Responsive Nanoparticles. Biomacromolecules.

[88] Kim B., Lee E., Kim Y., Park S., Khang G., Lee D. (2013). Dual Acid-Responsive Micelle-Forming Anticancer Polymers as New Anticancer Therapeutics. Adv. Funct. Mater..

[89] Raffai G., Kim B., Park S., Khang G., Lee D., Vanhoutte P.M. (2014). Cinnamaldehyde and cinnamaldehyde-containing micelles induce relaxation of isolated porcine coronary arteries: Role of nitric oxide and calcium. Int. J. Nanomed..

[90] Park S.-C., Kim N.-H., Yang W., Nah J.-W., Jang M.-K., Lee D. (2016). Polymeric micellar nanoplatforms for Fenton reaction as a new class of antibacterial agents. J. Control. Release.

[91] Xu J., Yan B., Du X., Xiong J., Zhou M., Wang H., Du Z. (2019). Acidity-triggered zwitterionic prodrug nano-carriers with AIE properties and amplification of oxidative stress for mitochondria-targeted cancer theranostics. Polym. Chem..

[92] Zhou J., Wang K., Jiang M., Li J., Yuan Y. (2022). Tumor-acidity and bioorthogonal chemistry-mediated construction and deconstruction of drug depots for ferroptosis under normoxia and hypoxia. Acta Biomater..

[93] Deng L., Feng Z., Deng H., Jiang Y., Song K., Shi Y., Liu S., Zhang J., Bai S., Qin Z. (2019). Rational Design of Nanoparticles to Overcome Poor Tumor Penetration and Hypoxia-Induced Chemotherapy Resistance: Combination of Optimizing Size and Self-Inducing High Level of Reactive Oxygen Species. ACS Appl. Mater. Interfaces.

[94] Qi X., Li N., Gu H., Xu Q., Li H., Ge J., Lu J. (2013). Preparation and self-assembly of a dual-functional copolymer for cancer therapy. React. Funct. Polym..

[95] Ma S., Song W., Xu Y., Si X., Lv S., Zhang Y., Tang Z., Chen X. (2020). Rationally Designed Polymer Conjugate for Tumor-Specific Amplification of Oxidative Stress and Boosting Antitumor Immunity. Nano Lett..

[96] Zhao X.Y., Shan P.F., Liu H.W., Li D.A., Cai P.H., Li Z.Y., Li Z.H. (2020). Poly(ethylene glycol)s With a Single Cinnamaldehyde Acetal Unit for Fabricating Acid-Degradable Hydrogel. Front. Chem..

[97] Hirose D., Kusuma S.B.W., Ina D., Wada N., Takahashi K. (2019). Direct one-step synthesis of a formally fully bio-based polymer from cellulose and cinnamon flavor. Green Chem..

[98] Manukumar H.M., Umesha S. (2017). Photocrosslinker technology: An antimicrobial efficacy of cinnamaldehyde cross-linked low-density polyethylene (Cin-C-LDPE) as a novel food wrapper. Food Res. Int..

[99] Dizdarevic A., Efiana N.A., Phan T.N.Q., Matuszczak B., Bernkop-Schnurch A. (2019). Imine bond formation: A novel concept to incorporate peptide drugs in self-emulsifying drug delivery systems (SEDDS). Eur. J. Pharm. Biopharm..

[100] Yu W., He X., Yang Z., Yang X., Xiao W., Liu R., Xie R., Qin L., Gao H. (2019). Sequentially responsive biomimetic nanoparticles with optimal size in combination with checkpoint blockade for cascade synergetic treatment of breast cancer and lung metastasis. Biomaterials.

[101] Luo S., Ma D., Wei R., Yao W., Pang X., Wang Y., Xu X., Wei X., Guo Y., Jiang X. (2022). A tumor microenvironment responsive nanoplatform with oxidative stress amplification for effective MRI-based visual tumor ferroptosis. Acta Biomater..

[102] Xu X., Zeng Z., Chen J., Huang B., Guan Z., Huang Y., Huang Z., Zhao C. (2020). Tumor-targeted supramolecular catalytic nanoreactor for synergistic chemo/chemodynamic therapy via oxidative stress amplification and cascaded Fenton reaction. Chem. Eng. J..

[103] (2020). Tien Canh LE. Extended Release Gastroretentive Formulation against Helicobacter Pylori. WO2020257936, 06/26, 2020. https://patentscope2.wipo.int/search/en/detail.jsf?docId=WO2020257936.

[104] Zhang Y., Li J., Pu K. (2022). Recent advances in dual- and multi-responsive nanomedicines for precision cancer therapy. Biomaterials.

